# Supplementary material for: Asthma Hospital Admission and Readmission Spikes, Advancing Accurate Classification to Advance Understanding of Causes
Source: Diagnostics (Basel). 2022 Oct 10;12(10):2445. doi: 10.3390/diagnostics12102445 (PMC9600648; doi:10.3390/diagnostics12102445)
Supplement: Supplementary file 1 [file diagnostics-12-02445-s001.zip › diagnostics-1912556-supplementary.pdf]

Supplement

File S1: Statistical Method—Details

We define robust as being resistant to outliers in the calculation of location and spread. We outline the algorithm used for the S-H-ESD method but detail, including extensive test results, is provided here [1]. This method

1. Uses a robust method for time series decomposition based on Locally Weighted Scatterplot Smoothing (LOESS) [2] to extract the seasonal component and is referred to as Seasonal and Trend decomposition using LOESS (STL) [3]. STL is made more robust by including a further weighting scheme [1]. See Figures S1 and S2.
2. In developing S-H-ESD, it was found that PPV was improved by extracting the median of the time series in the place of the time trend [1].
3. After extracting the seasonal component and median, the residuals are passed to the Rosner Extreme Studentized Test (ESD) [4]. The ESD uses a statistical test based on the null hypothesis that there are no outliers against the alternative that there are up to k outliers, where k is chosen by the user. The level of significance for the test is controlled by a Bonferroni adjustment dependent on k. The test iterates through the data, removing the found anomaly for the next iteration. K can be adjusted until beyond which no further outliers are detected and hence it is an exhaustive method. Statistical significance levels can be chosen as required.
4. The ESD was initially formulated using the mean and standard deviation but within the S-H-ESD approach, they are replaced by the median and median absolute deviation (MAD), robust measures of location and dispersion respectively [5]. Furthermore, as the

ESD was originally conceived as a test for outliers of a distribution that was approximately normal [4], the MAD is scaled by the 75th percentile of a standardized normal distribution, 1.4826, as a more robust estimate of dispersion irrespective of non-normality of the residuals [6].

File S2: Adapting the method for high asthma readmissions days (HARDs)

In applying this method, we found that it failed for the detection of HARDs in our data set. It detected that 39.4% of the readmissions were anomalous, a completely untenable result. The readmissions time series is an example of a low count time series. It had a range of 0 to 5 and the 60th, 75th and 95th percentiles were 0,1 & 2 respectively. More details are provided here [7]. Failure was due to a combination of the highly discrete nature of the time series due to its small range which was further dominated by zero. These factors combined to force over 98% of the time series residuals to be positive which gave the untenable result that 39.4% of the data were outliers, that is, basically all the non-zero readmission days.

To overcome this problem, we added random noise (jittered) from a uniform distribution between but not including -0.5 and 0.5 [U(-0.5, 0.5)]. Asymptotically, this has no effect on distribution on the mean of the readmissions time series 0.051, as the mean of two random variables is the sum of their means and the mean of a uniformly distributed variable between -0.5 and 0.5 is zero. The variance of the readmissions time series increases by the variance of U(-0.5, 0.5) which equals $^{1}/_{12}$, approximately equal to 0.083. The new variance is the sum of the variance of the non-jittered time series and $^{1}/_{12}$. It can be shown algebraically that this plays out to increase the

standard deviation of the original time series relatively by approximately 5.5%. This was corroborated by simulation experiments (10,000) which indicated that from a SD of .74, the jittered readmissions time series has a SD of 0.792 ± 0.004. The simulation experiments also indicated that the median for the jittered data would be expected to be about 0.31 ± 0.01 and the MAD 0.72 ± 0.01. We considered this median a suitable measure of location for the readmission time series as the mean of 0.51 in the unsmoothed data was pulled a little to the right due to 90% of its distribution being either 0 or 1, 9.5% was 2 or 3 and 17 observations (< 0.05%) had values of 4 or 5. The MAD for the jittered data closely emulated the SD of the non-jittered readmissions indicating that, as a measure of spread, it was little affected by the jittering. As the S.H.ESD uses the median and MAD, this indicates the robustness of this method to accurately capture location and spread and that adding smoothness did not negatively impact it.

The methods were implemented with freeware R [8]. S.H.ESD was implemented via the AnomalyDetection library [9] and a statistical significance level of p = 0.05 was nominated in classifying HAADs and HARDs. The R libraries ggplot [10] and stlplus [11] were used for graph plotting and times series decomposition respectively.

References

1. Hochenbaum J, Vallis OS, Kejariwal A. Automatic Anomaly Detection in the Cloud Via Statistical Learning. *ArXiv.* 2017;abs/1704.07706.
2. Cleveland WS. Robust Locally Weighted Regression and Smoothing Scatterplots. J Am Stat Assoc. 1979;74(368):829-36.
3. Robert BC, William SC, Irma T. STL: A Seasonal-Trend Decomposition Procedure Based on Loess. J. Off. Stat.1990;6(1):3.
4. Rosner B. Percentage Points for a Generalized ESD Many-Outlier Procedure. Technometrics. 1983; 25(2): 165-172.

5. Leys C, Ley C, Klein O, et al. Detecting outliers: Do not use standard deviation around the mean, use absolute deviation around the median. J. Exp. Soc. Psychol. 2013;49(4):764-6.
6. Rousseeuw PJ, Croux C. Alternatives to the Median Absolute Deviation. J Am Stat Assoc. 1993;88(424):1273-83.
7. Vicendese D, Olenko A, Dharmage S, et al. Modelling and predicting low count child asthma hospital readmissions using General Additive Models. Open J. Epidemiol. 2013;03:125-34.
8. R: The R Project for Statistical Computing [Internet]. R-project.org. 2022 [cited 16 May 2022]. Available from: https://www.R-project.org/
9. AnomalyDetection package - RDocumentation [Internet]. Rdocumentation.org. 2021 [cited 22 June 2021]. Available from: https://www.rdocumentation.org/packages/AnomalyDetection/versions/1.0
10. Create Elegant Data Visualisations Using the Grammar of Graphics [Internet]. Tidyverse.org. 2020. Available from: https://ggplot2.tidyverse.org.
11. Hafen R. stlplus: Enhanced Seasonal Decomposition of Time Series by Loess [Internet]. R-Packages. 2016 [cited 2022 May 16]. Available from: https://cran.r-project.org/web/packages/stlplus/index.html

Figure S1: Decomposition of all daily asthma hospital admissions using STL with the added robust scheme.

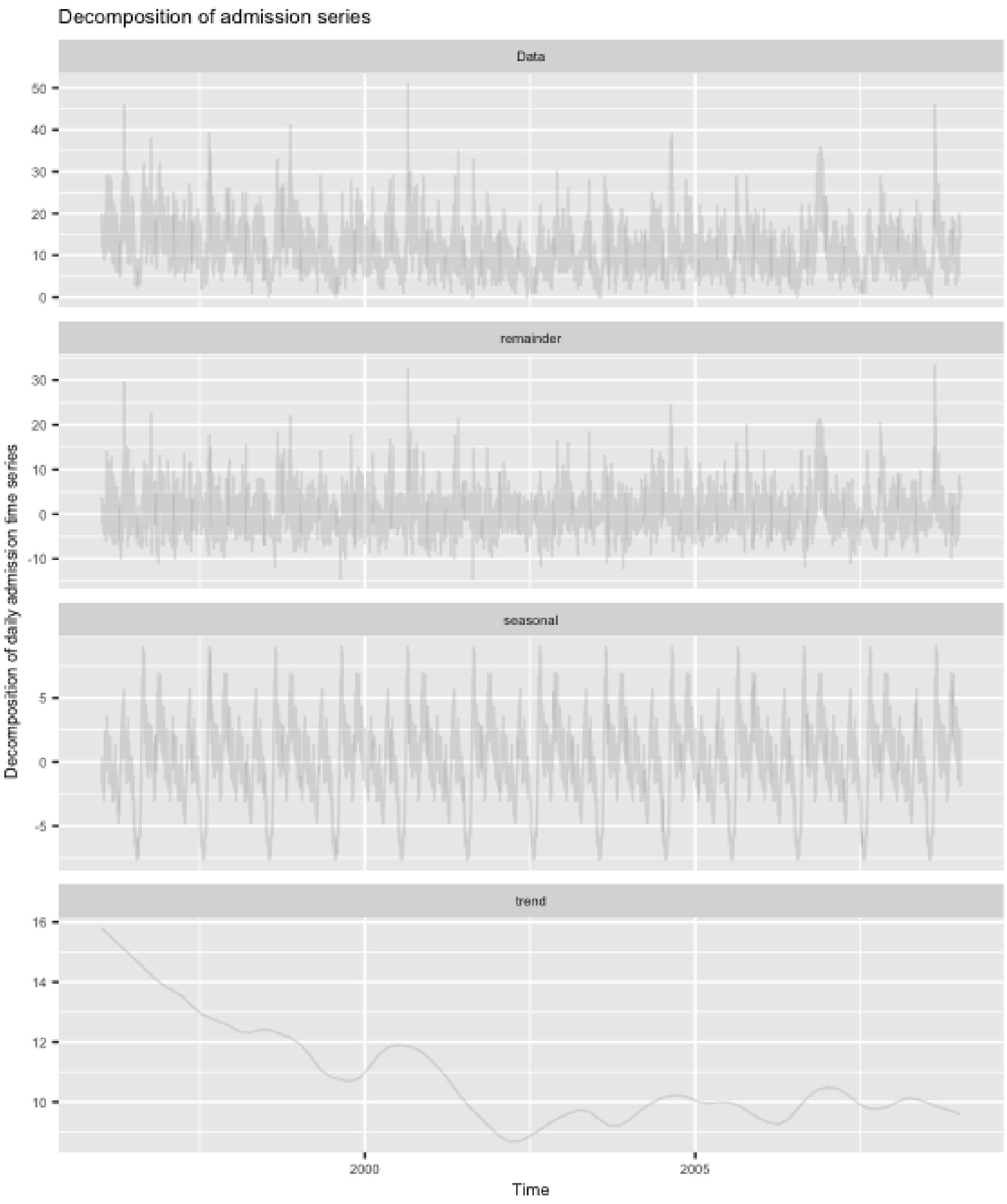

Figure S2: Decomposition of all daily asthma hospital readmissions using STL with the added robust scheme.

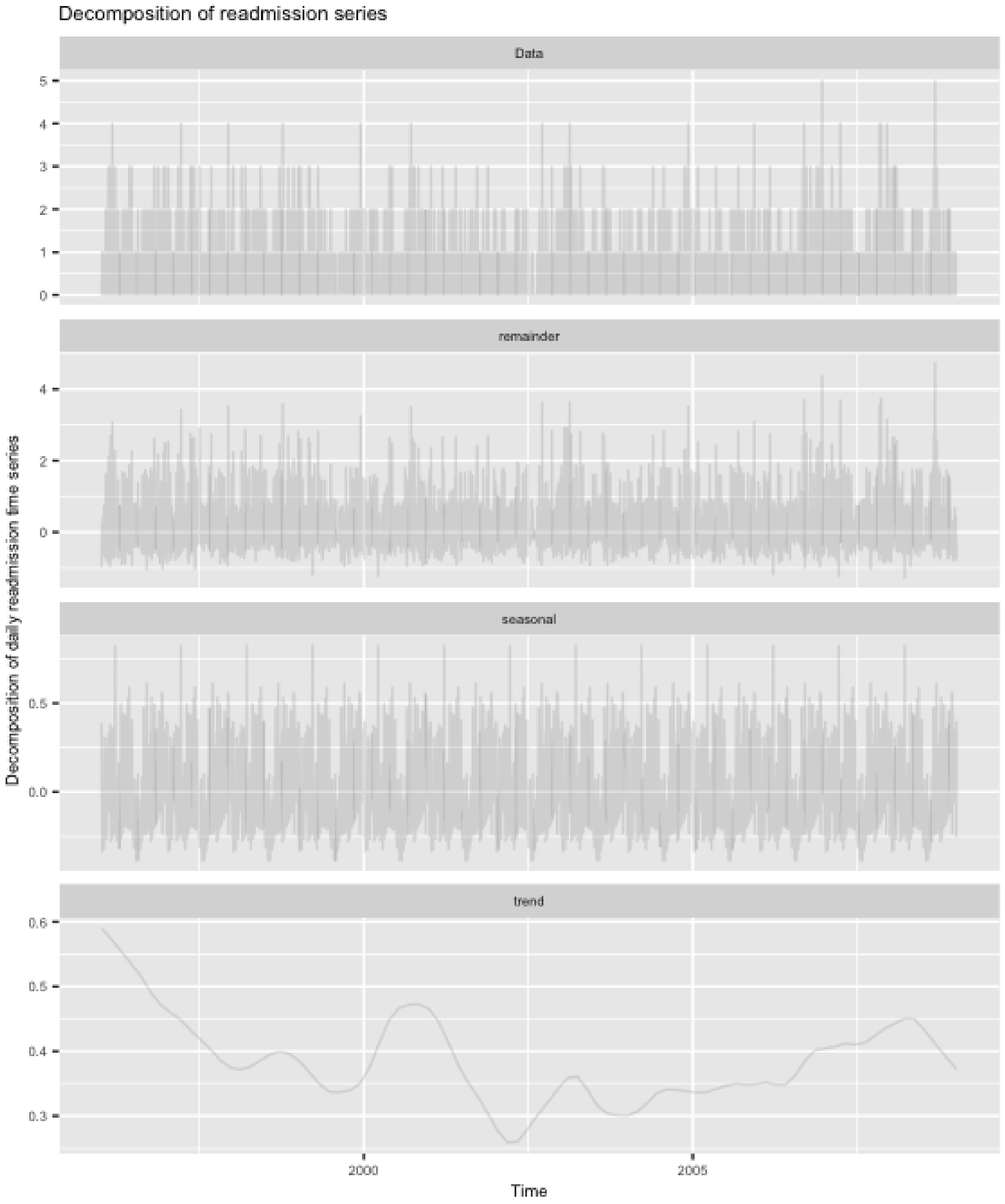

File S3: R Code for anomaly identification

```
# Written by Don Vicendese 2022 05 11      don.vicendese@unimelb.edu.au

# To download the S.H.ESD anomaly (outlier) detector.

# Just run the next 2 lines once only to install AnomalyDetection.

# Once installed, there is no need to run them again (unless re installing).

library(devtools)

devtools::install_github("twitter/AnomalyDetection")


# S.H.ESD was written by

# Hochenbaum J, Vallis OS, Kejariwal A. Automatic Anomaly Detection in the

 # Cloud Via Statistical Learning. ArXiv. 2017;abs/1704.07706.

# https://arxiv.org/abs/1704.07706


# Please read the help file.

library(AnomalyDetection)

help(AnomalyDetectionVec)

library(rio)                  # For file importing and exporting

library(ggplot2)      # for graphs

library(dplyr)            # For data manipulation


# Import your data
```

```
my.dat <-    import(put path and file name here)

# I assume the name of your time series is my.timeseries
# and it is in the data frame, my.dat

# Just to generalise a little
this.vec<- my.dat$my.timeseries

# Number of periods in your time series
# see https://otexts.com/fpp2/stl.html for details
# Say we have a 11 year time series and it acts in yearly cycles
my.period <- 11

# How many anomalies to search for.
# You can start at a low number and then work upwards
# until the number of anomalies does not change - max K,
# at the chosen alpha level of statistical evidence.
# Or start high and work down.
n_anom <-100

# This is a proportion of your time series length and
```

```
# determines up to how many anomalies you would like to detect.

my.max.anoms = n_anom/length(this.vec)


# Choose level of statistical significance.

my.alpha = 0.05


my.anom <-    AnomalyDetectionVec(this.vec , max_anoms = my.max.anoms,

                                   period = my.period, alpha = my.alpha,    e_value = TRUE,

                                    plot = TRUE, verbose = TRUE )


#This gives you the default graph.

my.anom$plot

# The plots can be embellished as required by passing my.anom$plot

# to ggplot and utilising all the ggplot functions as required.

# For example,

my.anom$plot + labs(y = my.ylabel, x = my.xlabel, title = my.title ) +

                                        scale_y_continuous(breaks = gg.breaks)

# Where my.ylabel etc. are defined by the user.

# See below for more graphing options.


# How many anomalies found.
```

```
length(my.anom$anoms$index)

# What were the values of the found anomalies.
my.anom$anoms$anoms
summary(my.anom$anoms$anoms)

# These are the data row numbers for the found anomalies.
my.anom$anoms$index

# The index can be used to identify the outliers in the original data set.
# For example,

# create a variable that = 1 if detected, 0 otherwise.
my.dat <- my.dat %>% mutate(detected = ifelse(row_number() %in%
                                        my.anom$anoms$index, 1, 0 ))
# Get a frequency table for detected.
with(my.dat, table(detected, useNA = "ifany"))

# Find the dates for these found high days.
# Assume the date variable is called date.
with(my.dat, date[detected == 1])
```

```
# The detected variable can be used to make a graph similar to above with
# my.anom$plot or other graphs as required. Here is an example.
ggplot(data = my.dat, aes(x = date , y =   my.timeseries) ) +   geom_line()   +
       geom_point(data = filter(my.dat, detected == 1), aes(x = date, y = my.timeseries) ,
   shape = 1, size = 3, colour = "red") + labs(y = gg.ylabel)   +
   scale_x_continuous(name = gg.xlabel,   breaks = gg.breaks,
                            labels = gg.labels)
# where date, gg.label etc are suitably defined.


# Get the help file.
       help(AnomalyDetectionVec)
```
